# Allele and genotype frequencies of the SOD1 c.118G>A mutation associated with degenerative myelopathy in Boxer and Pit Bull Terrier dogs from Uruguay

**DOI:** 10.29374/2527-2179.bjvm010525

**Published:** 2026-03-18

**Authors:** Rody Artigas, Carolina Menchaca, Eugenio Jara, Victoria Dávila, Alejandra Mondino, Noelia Vázquez, Silvia Llambí

**Affiliations:** 1 Unidad académica de Mejora Animal. Departamento de Producción Animal y Salud de los Sistemas Productivos. Facultad de Veterinaria, Udelar. Montevideo, Uruguay.; 2 Centro de Encefalopatías y Enfermedades Emergentes Transmisibles. Unizar, Zaragoza, España.; 3 Department of Clinical Scinces, North Carolina State University, North Carolina, United States.; 4 Unidad académica de Anatomía, Departamento de Biociencias, Facultad de Veterinaria, Udelar, Montevideo, Uruguay.

**Keywords:** boxer, degenerative myelopathy, genetic disease, pit bull terrier, SOD1 gene, boxer, mielopatia degenerativa, doença genética, pit bull terrier, gene SOD1

## Abstract

Canine degenerative myelopathy (DM) is a hereditary disease of the spinal cord, characterized by a progressive clinical course, and the death of affected animals. This study aimed to investigate the population distribution of the SODc.118:G˃A mutation responsible for the disease. For this purpose, 131 dogs from both breeds (Pit Bull Terrier, n=64, and Boxer, n=67) were studied. Genomic DNA was extracted from peripheral blood, and the mutation was identified using the PCR-RFLP technique. For each population, the mutant allele frequency, genotypic frequency, and Hardy–Weinberg equilibrium were calculated. The frequency of the DM-related A allele was 0.16 in the Pit Bull Terrier and 0.52 in the Boxer breed. Both populations were in Hardy–Weinberg equilibrium (p > 0.05). The genotypic distribution differed between the two breeds analyzed and also between different populations within each breed (p < 0.05). Simulation of the evolution of the mutant allele frequency under different genetic drift models showed that the variance of the allele frequency increased as the effective population size (Ne) decreased. The frequency detected in the Boxer breed was high, and although it was lower in the Pit Bull Terrier, the allele was still present. These findings support the potential use of genotyping tests for degenerative myelopathy management and provide descriptive population data from dogs of both breeds in Uruguay.

## Resumo

A mielopatia degenerativa canina (MD) é uma doença hereditária da medula espinhal, caracterizada por um curso clínico progressivo e pela morte dos animais afetados. Este estudo teve como objetivo investigar a distribuição populacional da mutação SODc.118:G˃A responsável pela doença. Para isso, foram estudados 131 cães de ambas as raças (Pit Bull Terrier, n=64, e Boxer, n=67). O DNA genômico foi extraído do sangue periférico e a mutação foi identificada usando a técnica PCR-RFLP. Para cada população, foram calculadas a frequência do alelo mutante, a frequência genotípica e o equilíbrio de Hardy-Weinberg. A frequência do alelo A relacionado à DM foi de 0,16 na raça Pit Bull Terrier e de 0,52 na raça Boxer. Ambas as populações estavam em equilíbrio de Hardy-Weinberg (p > 0,05). A distribuição genotípica diferiu entre as duas raças analisadas e também entre diferentes populações dentro de cada raça (p < 0,05). A simulação da evolução da frequência do alelo mutante sob diferentes modelos de deriva genética mostrou que a variância da frequência do alelo aumentou à medida que o tamanho efetivo da população (Ne) diminuiu. A frequência detectada na raça Boxer foi alta e, embora fosse menor no Pit Bull Terrier, o alelo ainda estava presente. Esses achados apoiam o uso potencial de testes de genotipagem para o manejo da mielopatia degenerativa e fornecem dados descritivos da população de cães de ambas as raças no Uruguai

## Introduction

Canine degenerative myelopathy (DM) (OMIA 000263-9615) is an inherited disease of the spinal cord, characterized by its progressive course and fatal outcome, for which no specific treatment is currently available (Kathmann et al., 2006). Clinical signs include proprioceptive ataxia and spastic paralysis of the pelvic limbs, eventually progressing to paraplegia, with loss of sphincter control and, in advanced stages, tetraplegia (Coates et al., 2007; Coates & Wininger, 2010; Dewey & Costa, 2016).

The onset of DM varies among breeds but typically begins around 5 years of age (Coates & Wininger, 2010). The disease progresses from the thoracolumbar spinal cord and may lead to non-ambulatory paraparesis and early euthanasia in large breeds (Coates et al., 2007; Nardone et al., 2016).

The inheritance pattern of DM is autosomal recessive with incomplete penetrance. Mutations associated with the disease are located in the SOD1 gene, which encodes the enzyme superoxide dismutase 1 (Wininger et al., 2011; Awano et al., 2009). This enzyme plays a key role in maintaining oxidative homeostasis in the central nervous system by converting superoxide radicals into hydrogen peroxide and oxygen (Castillo et al., 2014).

The first reported mutation in SOD1 is a G>A substitution (c.118G>A) in exon 2, resulting in the replacement of glutamine with lysine at residue 40 of the protein (E40K) (Awano et al., 2009). This mutation is considered ancestral, as it has been found in several breeds, including mixed-breed dogs (Zeng et al., 2014). The second mutation is an A>T transversion (c.52A>T) in exon 1 of SOD1, resulting in a threonine-to-serine substitution at residue 18 (T18S) of the protein. Unlike the previous one, this mutation is unique to the Bernese Mountain Dog (Wininger et al., 2011).

Definitive diagnosis of degenerative myelopathy can only be achieved post mortem through immunohistochemical detection of mutant SOD1 aggregates (Awano et al., 2009; Zeng et al., 2014). Consequently, antemortem diagnosis relies on the exclusion of other causes and on molecular genotyping, which represents a key tool for disease assessment in living animals (Coates & Wininger, 2010; Bouché et al., 2023).

Currently, the c.118G>A mutation has been reported in more than 60 dog breeds (Nicholas & Tammen, 2024); however, its population dynamics have been evaluated in only a limited number of populations. The aim of this study was to investigate the frequency of the c.118G>A mutation associated with DM in Pit Bull Terrier and Boxer dogs in Uruguay and to place these results in an international context.

## Materials and methods

### Ethical approval and informed consent

This study was approved by the Commission on Ethics in the Use of Animals (CEUA) (approval nos. 1512 and 1264). Samples were obtained with written informed consent from the owners.

### Study period and location

The study was conducted from March 2023 to March 2025 at the Facultad de Veterinaria, Udelar, Montevideo, Uruguay.

### Animals

Peripheral whole blood samples were collected in EDTA tubes from 131 dogs of the breeds Pit Bull Terrier (n=64) and Boxer (n=67), from different regions of Uruguay. The animals included both sexes, were of different ages, were not closely related, and did not present clinical signs compatible with DM.

### DNA extraction and genotyping of the c.118G>A mutation

Genomic DNA was extracted from 200 μL of whole blood using the Quick-DNA Miniprep Kit (Zymo Research, USA) according to the manufacturer’s instructions. The DNA obtained was quantified using a DeNovix DS11 spectrophotometer (DeNovix Inc., USA). Samples were standardized to a final concentration of 50 ng/μL.

Genotyping for the c.118G>A mutation was performed using the PCR-RFLP technique. Primers previously designed by Holder et al. (2014) were used to amplify a 292 bp fragment of the SOD1 gene containing the mutation. Amplification was carried out using a Multigene II thermal cycler (Labnet Inc., USA) in a final reaction volume of 25 μL containing 50 ng of genomic DNA, 1 μL of each primer (10 pmol/μL), 12.5 μL of ImmoMix (Bioline, Australia), and Milli-Q water to complete the volume. The amplification program consisted of an initial denaturation at 95°C for 10 min, followed by 35 cycles of 94°C for 40 s (denaturation), 55°C for 30 s (annealing), and 72°C for 1 min (extension), with a final extension at 72°C for 10 min. The amplicons were verified by 1.5% agarose gel electrophoresis using GoodView™ (SBS Genetech Co., Ltd., China) as a stain.

The amplicons were digested with the AcuI restriction enzyme. The reaction was performed in a final volume of 25 μL containing 2.5 μL of digestion buffer (NEB, New England Biolabs, USA), 11.5 μL of ultrapure water, 1 μL of AcuI enzyme (NEB, New England Biolabs), and 10 μL of PCR product. The reaction was incubated overnight at 37°C and inactivated for 20 min at 65°C. Genotypes were determined by analyzing the digestion products using 1.5% agarose gel electrophoresis stained with GoodView™ (SBS Genetech Co., Ltd.). RFLP validation had been previously performed in our laboratory (Artigas et al., 2024).

### Population analysis

Allele frequencies, genotypic frequencies, and Hardy–Weinberg equilibrium were calculated using Fisher’s exact test in the program Genepop 4.2, available at Genepop (2026).

### Analysis of allele frequencies under genetic drift in the Boxer breed

Simulation of the frequency of the c.118G>A mutation under genetic drift in the Boxer breed in Uruguay was carried out, using the frequency detected in this study as representative of the population. Since no previous calculations of genetic diversity or estimates of Ne (effective population size) are available at the national level, different models were applied. According to information obtained from the Uruguayan Kennel Club, approximately 500 animals have been registered in the last 8 years. Given the difficulties in calculating Ne, it was assumed that approximately 10% of the registered animals are used for breeding (Ukawa et al., 2024), resulting in a first estimate of Ne = 50. Considering that 20% of the breeding animals are males and 80% are females, the minimum Ne was estimated as 32 using Wright’s formula (Wright, 1931):


$$Ne=\frac{4NmNf}{Nm+Nf}$$ (1)

Additionally, two theoretical scenarios were considered: one with Ne = 113, based on previous genealogical studies in the breed (Mostert et al., 2015), and another with a higher hypothetical number Ne = 200. Model simulations were performed using 1locussim software (Carvajal-Rodríguez, 2023b) following Carvajal-Rodríguez (2023a), considering a maximum of 100 populations and 100 generations.

## Results

**Figure 1:**
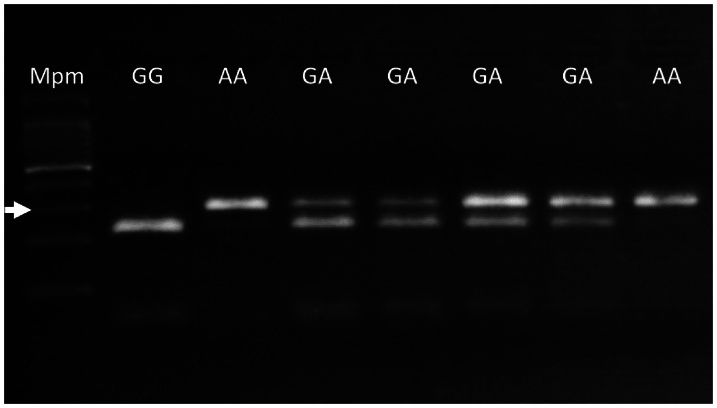
1.5% agarose gel electrophoresis of digestion patterns with the AcuI enzyme for the SOD1:c118 G>A site. GG: dominant homozygous genotype; GA: heterozygous genotype; AA: recessive homozygous genotype related to an increased risk of degenerative myelopathy. Mpm: 100bp molecular weight marker. The white arrow indicates the 300bp line of the Mpm.

PCR amplification yielded a 292 bp amplicon, which, when digested with the AcuI enzyme, produced three distinct banding patterns. Animals homozygous for the mutation (AA) exhibited a single 292 bp band, heterozygotes (GA) exhibited three bands (292 bp, 230 bp, and 62 bp), and normal homozygotes (GG) exhibited two bands (230 bp and 62 bp) (Figure 1).

Allele and genotypic frequencies are shown in Table 1. Both populations were in Hardy–Weinberg equilibrium. Comparison between the two breeds revealed a significant difference in genotype distribution (Table 1; p < 0.00001). Similarly, the distribution of genotypes in the Boxer population in Uruguay differed from that reported in the United States and Colombia (p < 0.05; Table 2). For the Pit Bull Terrier breed, the genotypic distribution also differed from that reported in the United States (p < 0.00001; Table 3).

**Table 1:** Allelic and genotypic frequencies for the c.118G>A mutation of the SOD1 gene in Pit Bull Terrier and Boxer dogs from Uruguay.

**Genotype**	**Pit Bull Terrier (N=64)**		**Bóxer (N=67)**		***p* value** *
	**Observed**	**Expected**	**Observed**	**Expected**	
GG	45 (0.7)	45 (0.7)	13 (0.19)	15 (0.22)	<0.00001
GA	17 (0.27)	17 (0.27)	38 (0.57)	32 (0.48)	---
AA	2 (0.03)	2 (0.03)	16 (0.24)	20 (0.3)	---
**Allele**	**Pit Bull Terrier**		**Bóxer**		**HWE p value**
G	0.84		0.47		0.47
A	0.16		0.52		1

* p value corresponds to the comparison in the distribution of genotypes between breeds.

**Table 2:** Genotypic frequencies for the SOD1 c.118G>A mutation in different populations of Boxer dogs internationally.

**Country**	**N**	**Genotype**			**P value** *	**Reference**
		**GG**	**GA**	**AA**		
USA	3934	500 (0.13)	1177 (0.3)	2257 (0.57)	<0.00001	Zeng et al. (2014)
USA	157	14 (0.09)	57 (0.36)	86 (0.55)	<0.0001	Awano et al. (2009)
Colombia	62	34 (0.55)	21 (0.34)	7 (0.11)	<0.001	Ayala-Valdovinos et al. (2018)
Uruguay	67	13 (0.19)	38 (0.57)	16 (0.24)	----	This Study

* p value corresponds to the comparison in the distribution of genotypes between the Boxer population of other countries and Uruguay.

**Table 3:** Genotypic frequencies for the SOD1 c.118G>A mutation in two populations of Pit Bull Terrier dogs.

**Country**	**N**	**Genotype**			P value*	**Reference**
		**GG**	**GA**	**AA**		
USA	53	23 (0.43)	6 (0.11)	24 (0.45)	<0.00001	Zeng et al. (2014)
Uruguay	64	45 (0.7)	17 (0.27)	2 (0.03)	----	This Study

Simulation of the c.118G>A mutation frequency under the four genetic drift models considered showed that the mean allele frequencies remained similar across models. However, the variance of the allele frequency was higher in the model with Ne = 32 than in the other models (Ne = 50, Ne = 113, and Ne = 200).

## Discussion

The present study reports the presence of the SOD1 c.118G>A mutation in Boxer and Pit Bull Terrier dogs from Uruguay. The frequency of the A allele was higher in the Boxer population than in the Pit Bull Terrier population (Table 1).

When analyzing the Boxer breed in an international context, the distribution of genotypes among populations was significantly different from that observed in Uruguay (Table 1). These differences may be explained by founder effects, genetic drift, or by mating selection decisions in each country.

The frequency of the mutant allele in the Boxer population of Uruguay was higher than that reported in Colombia and lower than values historically reported in the United States (Ayala-Valdovinos et al., 2018; Zeng et al., 2014; Awano et al., 2009). More recent data indicate a decrease in allele frequency in the United States, although it remains elevated, possibly reflecting the availability of genetic testing and more informed breeding decisions (Donner et al., 2023). For the Pit Bull Terrier breed, a significantly lower allele frequency was observed in Uruguay (Table 3) compared with reports from the United States (Zeng et al., 2014).

International variability in the frequency of the SOD1 c.118G>A mutation across dog breeds is summarized in Supplementary Table S1. Allele frequencies range from very low to near fixation, supporting the ancestral nature of the mutation and highlighting substantial heterogeneity among breeds and populations (Zeng et al., 2014; Donner et al., 2023).

Both populations studied in Uruguay were in Hardy–Weinberg equilibrium (p > 0.05) for the SOD1 c.118G>A mutation, consistent with the late onset and incomplete penetrance of the disease, as well as the absence of genotype based selection programs in the studied populations (Capucchio et al., 2014).

The high mutation frequency in the Boxer population and its presence in Pit Bull Terriers in Uruguay demonstrate the need for disease control programs. These programs should be applied cautiously to avoid population bottlenecks, reduced genetic variability, and inbreeding depression (Mataragka et al., 2021; Santos et al., 2020).

**Figure 2:**
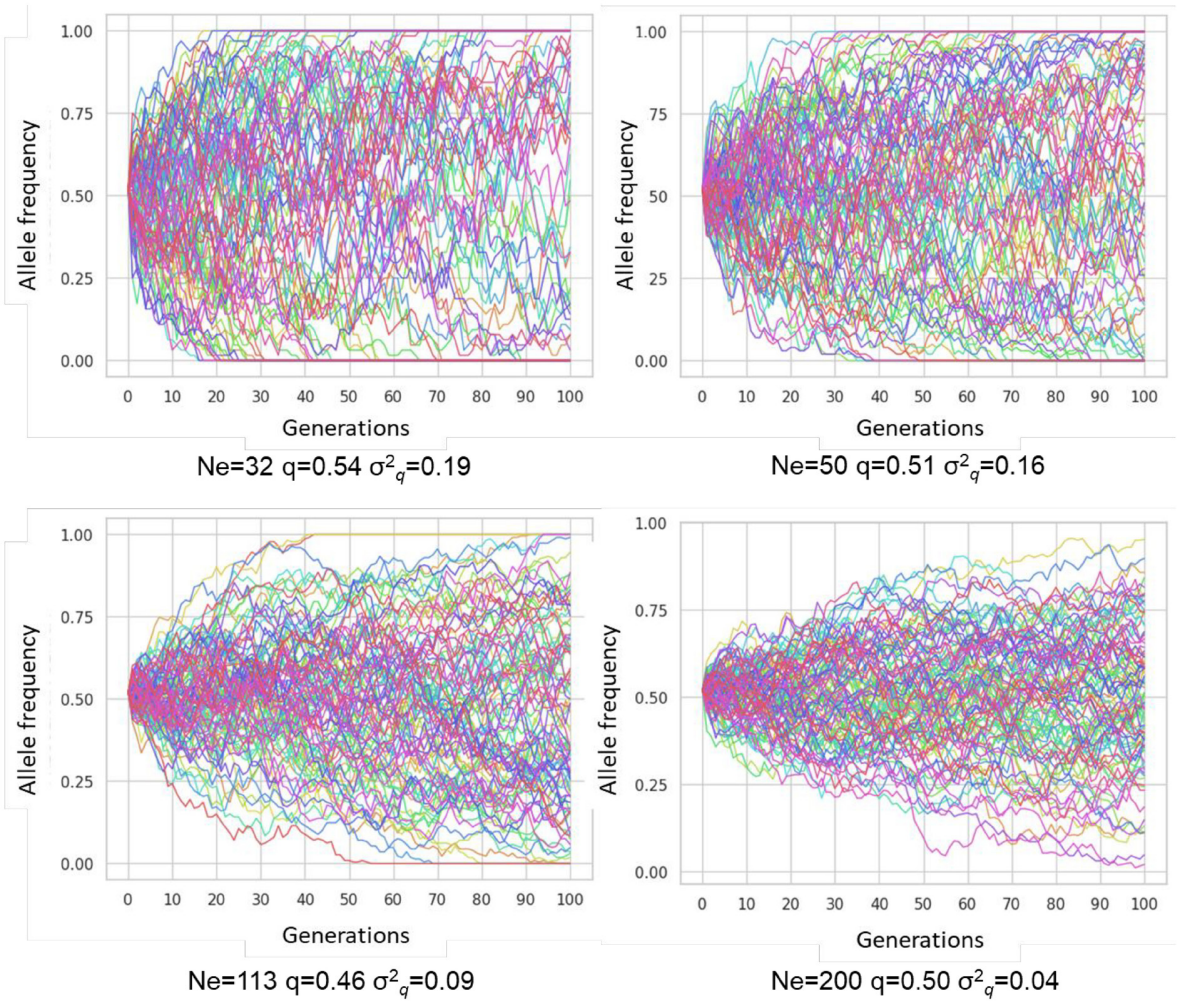
Simulation of the allelic frequency of the SOD1:c118 G>A mutation in the Uruguay Boxer breed over generations under the effect of genetic drift.

Simulation of the evolution of the SOD1 c.118G>A mutation over 100 generations showed that, in the absence of selection, the mean allele frequency remains relatively stable across models (0.46–0.54). However, variance was higher in the Ne = 32 model (Figure 2), suggesting that allele fixation could occur earlier in populations with small effective size. Therefore, in breeds with a small census and high DM susceptibility, introducing non-carrier animals is important to reduce allele frequency, increase Ne, and mitigate inbreeding depression.

Responsible breeding strategies, such as crossing with GG animals, can improve genetic variability in the short term, especially when using animals from different families or countries. In breeds where the allele frequency is near fixation or very high, as in the Wire Fox Terrier (0.82–0.94; Donner et al., 2023; Zeng et al., 2014), this strategy may be less effective due to the risk of overusing a few breeders.

In Japan, the Pembroke Welsh Corgi population demonstrated that the frequency of the mutant allele can be reduced from 39.4% to 2.9% in 5 years with a higher Ne at the end of the period (Ukawa et al., 2024). This shows that high directional selection can be applied without significant loss of genetic variability if selective matings include animals from other lineages, families, or countries.

Because DM manifests late, the effects of genotypic control programs are expected in the long term, typically 5–8 years after implementation, corresponding to the time of symptom onset (Donner et al., 2023). Further studies including additional populations, breeds, and larger sample sizes will be required to expand these findings.

## Conclusions

This study reports the presence of the SOD1 c.118G>A mutation in Boxer and Pit Bull Terrier dogs in Uruguay. The frequency detected in the Boxer breed is high, and although it is lower in the Pit Bull Terrier, the allele is still present. These results support the potential use of genotyping tests in the context of future discussions on DM management, applying the most efficient crossbreeding strategies for each breed.

## Supplementary Material

Supplementary material accompanies this paper.

Table S1 Frequency of the SOD1 c.118G>A mutation in different dog breeds worldwide.

This material is available as part of the online article from https://doi.org/10.29374/2527-2179.bjvm010525
