# Supplementary material for: Allele and genotype frequencies of the SOD1 c.118G>A mutation associated with degenerative myelopathy in Boxer and Pit Bull Terrier dogs from Uruguay
Source: Braz J Vet Med. 2026 Mar 18;48:e010525. doi: 10.29374/2527-2179.bjvm010525 (PMC13002109; doi:10.29374/2527-2179.bjvm010525)
Supplement: Table S1 [file bjvm-48-e010525-suppl01.pdf]

Table S1. Frequency of the SOD1 c.118G>A mutation in different dog breeds worldwide.

Breed	Country	N	A Freq.	Reference
Airedale Terrier	USA	200	0.1275	Donner et al. [20]
Alaskan Malamute	USA	61	0.02	Zeng et al. [10]
Alaskan Malamute	USA	504	0.0009	Donner et al. [20]
American Eskimo Dog	USA	302	0.23	Donner et al. [20]
American Foxhound	USA	574	0.04	Donner et al. [20]
American Staffordshire Terr.	USA	42790	0,064	Donner et al. [20]
American Water Spaniel	USA	91	0.51	Zeng et al. [10]
Anatolian Shepherd Dog	USA	66	0.075	Donner et al. [20]
Australian Cattle Dog	USA	61	0.09	Zeng et al. [10]
Australian Cattle Dog	USA	982	0.05	Donner et al. [20]
Australian Kelpie	USA	104	0.03	Donner et al. [20]
Australian Shepherd	USA	113	0.41	Zeng et al. [10]
Australian Shepherd	Italiy	154	0.07	Ghilardi et al [24]
Australian Shepherd	USA	2296	0.09	Donner et al. [20]
Australian Shepherd	France	198	0.1	Majchrakova et al. [25]
Australian Shepherd	Germany	123	0.13	Majchrakova et al. [25]
Australian Shepherd	Czech Rep.	105	0.14	Majchrakova et al. [25]
Australian Shepherd	Beilgium	88	0.11	Majchrakova et al. [25]
Australian Shepherd	Austria	55	0.11	Majchrakova et al. [25]
Basset Hound	USA	990	0.0025	Donner et al. [20]
Beagle	USA	57	0.06	Zeng et al. [10]

Beagle	USA	5292	0.017	Donner et al. [20]
Belgian Malinois	Greece	68	0.029	Kountourantzis et al. [26]
Belgian Malinois	USA	101	0.06	Zeng et al. [10]
Belgian Malinois	Greece	72	0.17	Mataragka et al. [22]
Belgian Malinois	USA	1186	0.042	Donner et al. [20]
Bernese Mountain Dog	USA	2413	0.38	Zeng et al. [10]
Bernese Mountain dog	Italy	129	0.17	Ghilardi et al [24]
Bernese Mountain Dog	USA	955	0.26	Donner et al. [20]
Bichon Frise	USA	1069	0.026	Donner et al. [20]
Biewer Terrier	USA	184	0.15	Donner et al. [20]
Bloodhound	USA	264	0.30	Zeng et al. [10]
Bloodhound	USA	280	0.225	Donner et al. [20]
Border Coliie	Colombia	80	0.01	Ayala-Valdovinos et al. [19]
Border Collie	USA	80	0.17	Zeng et al. [10]
Border Collie	USA	6714	0.003	Donner et al. [20]
Borzoi	USA	787	0.17	Zeng et al. [10]
Boston terrier	USA	3702	0.078	Donner et al. [20]
Boxer	USA	3934	0.72	Zeng et al. [10]
Boxer	USA	157	0.72	Awano et al. [8]
Boxer	USA	4552	0.42	Donner et al. [20]
Boxer	Colombia	62	0.28	Ayala-Valdovinos et al. [19]
Boykin Spaniel	USA	154	0.14	Donner et al. [20]
Brittany	USA	349	0.001	Donner et al. [20]
Bull Terrier (Standard)	USA	468	0,005	Donner et al. [20]
Bulldog (American)	USA	540	0,03	Donner et al. [20]

Bulldog (Standard)	USA	4816	0,13	Donner et al. [20]
Bullmastiff	USA	209	0,02	Donner et al. [20]
Cairn Terrier	USA	183	0,027	Donner et al. [20]
Canaan Dog	USA	169	0.29	Zeng et al. [10]
Cane Corso	USA	145	0,021	Donner et al. [20]
Cardigan Welsh Corgi	USA	544	0.32	Zeng et al. [10]
Cardigan Welsh Corgi	USA	125	0,2	Donner et al. [20]
Catahoula Leopard Dog	USA	154	0,07	Donner et al. [20]
Cavalier King Charles Spaniel	USA	73	0.68	Zeng et al. [10]
Cavalier King Charles Spaniel	USA	2242	0,51	Donner et al. [20]
Chesapeake Bay Retriever	USA	2344	0.37	Zeng et al. [10]
Chesapeake Bay Retriever	USA	138	0,25	Donner et al. [20]
Chihuahua	USA	4273	0,015	Donner et al. [20]
Chinese Crested	USA	53	0.12	Zeng et al. [10]
Chinese Crested	USA	204	0,06	Donner et al. [20]
Chinese Shar-Pei	USA	400	0,004	Donner et al. [20]
Chow Chow	USA	643	0,075	Donner et al. [20]
Cocker Spaniel	USA	1881	0,0019	Donner et al. [20]
Collie	USA	151	0.39	Zeng et al. [10]
Collie	USA	1207	0,13	Donner et al. [20]
Coton de Tulear	USA	110	0.07	Zeng et al. [10]
Coton de Tulear	USA	104	0,024	Donner et al. [20]
Czechoslovakian Wolfdog	USA	52	0.34	Zeng et al. [10]
Czechoslovakian wolfdog	Italy	206	0.17	Ghilardi et al [24]
Dachshund (Miniature Longhaired)	USA	213	0,0047	Donner et al. [20]

Dachshund (Miniature Shorthaired)	USA	585	0,006	Donner et al. [20]
Dalmatian	USA	180	0.03	Zeng et al. [10]
Dalmatian	USA	820	0,024	Donner et al. [20]
Danish Swedish Farmdog	USA	61	0,033	Donner et al. [20]
Doberman Pinscher	USA	55	0.02	Zeng et al. [10]
Dobermann Pinscher	USA	2219	0,0045	Donner et al. [20]
Dogo Argentino	USA	225	0.004	Donner et al. [20]
English Cocker Spaniel	USA	580	0.0009	Donner et al. [20]
English Springer Spaniel	USA	127	0.17	Zeng et al. [10]
English Springer Spaniel	USA	751	0,034	Donner et al. [20]
Finnish Lapphund	USA	57	0,15	Donner et al. [20]
Fox Terrier (Wire)	USA	182	0,82	Donner et al. [20]
French Bulldog	USA	87	0.16	Zeng et al. [10]
French bulldog	Italy	133	0.22	Ghilardi et al [24]
French Bulldog	USA	13111	0,2	Donner et al. [20]
German Sheperherd	UK	150	0.377	Holder et al. [14]
German Sheperherd	USA	6458	0.366	Zeng et al. [10]
German Sheperherd	USA	83	0.331	Shaffer et al. [27]
German Sheperherd	Italy	47	0.170	Capucchio et al. [21]
German Sheperherd	Poland	47	0.181	Shaffer et al. [27]
German Sheperherd	Israel	20	0.175	Shaffer et al. [27]
German Sheperherd	México	31	0.130	Ayala Valdovinos et al. [19]
German Sheperherd	Brazil	96	0.121	Santos et al. [23]
German Sheperherd	Japan	541	0.220	Maki et al. [28]
German Sheperherd	Romania	40	0.20	Cocostîrc et al. [29]

German Sheperherd	Greece	29	0.138	Kountourantzis et al. [26]
German Sheperherd	Uruguay	114	0.23	Artigas et al. [30]
German Sheperherd	Paraguay	44	0.15	Artigas et al. [30]
German Sheperherd	Italy	265	0.29	Ghilardi et al [24]
German Shepherd Dog	USA	15645	0,2	Donner et al. [20]
German Shorthaired Pointer	USA	1252	0,025	Donner et al. [20]
German Wirehaired Pointer	USA	84	0,042	Donner et al. [20]
Golden Retriever	USA	334	0.03	Zeng et al. [10]
Golden Retriever	USA	12881	0,0017	Donner et al. [20]
Great Dane	USA	3266	0,0014	Donner et al. [20]
Great Pyrenees	USA	85	0.14	Zeng et al. [10]
Great Pyrenees	USA	1985	0,083	Donner et al. [20]
Greyhound	USA	105	0,0048	Donner et al. [20]
Havanese	USA	441	0,0011	Donner et al. [20]
Hovawart	USA	64	0.38	Zeng et al. [10]
Hovawart	Italy	37	0.28	Ghilardi et al [24]
Irish Setter	USA	57	0.16	Zeng et al. [10]
Irish Setter	USA	83	0,024	Donner et al. [20]
Irish Wolfhound	USA	78	0,0064	Donner et al. [20]
Jack Russell Terrier	USA	60	0.32	Zeng et al. [10]
Japanese Shiba Inu	USA	2123	0,0012	Donner et al. [20]
Kerry Blue Terrier	USA	558	0.34	Zeng et al. [10]
Komondor	USA	55	0.22	Zeng et al. [10]
Kooikerhondje	USA	200	0,0025	Donner et al. [20]
Kuvasz	USA	75	0.26	Zeng et al. [10]

Labrador Retriever	USA	475	0.07	Zeng et al. [10]
Labrador Retriever	USA	16855	0,0058	Donner et al. [20]
Lagotto Romagnolo	USA	623	0,0016	Donner et al. [20]
Lhasa Apso	USA	243	0,0082	Donner et al. [20]
Maltese	USA	2413	0,0054	Donner et al. [20]
Mastiff	USA	767	0,084	Donner et al. [20]
Mastiff (English Mastif)	USA	114	0.10	Zeng et al. [10]
Miniature American Shepherd	USA	1476	0,037	Donner et al. [20]
Miniature Pinscher	USA	658	0,11	Donner et al. [20]
Mixed Breed	USA	81159<br>1	0,075	Donner et al. [20]
Neapolitan Mastiff	USA	90	0,028	Donner et al. [20]
Newfoundland	USA	62	0.02	Zeng et al. [10]
Newfoundland	USA	463	0,01	Donner et al. [20]
Norwich Terrier	USA	74	0.09	Zeng et al. [10]
Nova Scotia Duck Tolling Ret.	USA	59	0.07	Zeng et al. [10]
Nova Scotia Duck Tolling Ret.	USA	63	0,048	Donner et al. [20]
Old English Sheepdog	USA	423	0,0012	Donner et al. [20]
Papillon	USA	197	0,038	Donner et al. [20]
Parson Russell Terrier	USA	181	0,14	Donner et al. [20]
Pekingese	USA	239	0,012	Donner et al. [20]
Pembroke Welsh Corgi	Japan	122	0.697	Chang et al. [31]
Pembroke Welsh Corgi	USA	3209	0.79	Zeng et al. [10]
Pembroke Welsh corgi	USA	67	0.76	Awano et al. [8]
Pembroke Welsh Corgi	Italy	91	0.55	Ghilardi et al [24]
Pembroke Welsh Corgi	USA	4364	0,53	Donner et al. [20]

Pit Bull Terrier	USA	53	0.51	Zeng et al. [10]
Pomeranian	USA	5294	0,09	Donner et al. [20]
Poodle	Italy	174	0.06	Ghilardi et al [24]
Poodle (Miniature)	USA	3555	0,049	Donner et al. [20]
Poodle (Standard)	USA	4203	0,038	Donner et al. [20]
Poodle (Toy)	USA	94	0,005	Donner et al. [20]
Poodle-Standard	USA	533	0.07	Zeng et al. [10]
Presa Canario	USA	64	0,094	Donner et al. [20]
Pug	USA	382	0.32	Zeng et al. [10]
Pug	USA	5154	0,26	Donner et al. [20]
Puli	USA	123	0.17	Zeng et al. [10]
Pumi	USA	86	0,0059	Donner et al. [20]
Rat Terrier	USA	63	0.01	Zeng et al. [10]
Rhodesian Ridgeback	USA	2645	0.28	Zeng et al. [10]
Rhodesian Ridgeback	USA	323	0,054	Donner et al. [20]
Rottweiler	USA	76	0.03	Zeng et al. [10]
Rottweiler	USA	4718	0,006	Donner et al. [20]
Russell Terrier	USA	239	0,06	Donner et al. [20]
Saarloos Wolfdog	USA	95	0,09	Donner et al. [20]
Saint Bernard	USA	78	0.13	Zeng et al. [10]
Saint Bernard	USA	721	0,012	Donner et al. [20]
Samoyed	USA	550	0,0018	Donner et al. [20]
Schnauzer (Giant)	USA	230	0,0043	Donner et al. [20]
Schnauzer (Miniature)	USA	4638	0,0027	Donner et al. [20]
Scotch collie	Italy	33	0.36	Ghilardi et al [24]

Shetland Sheepdog	USA	58	0.21	Zeng et al. [10]
Shetland Sheepdog	USA	945	0,09	Donner et al. [20]
Shih Tzu	USA	7527	0,17	Donner et al. [20]
Shiloh Shepherd	USA	221	0.15	Zeng et al. [10]
Siberian Husky	USA	97	0.04	Zeng et al. [10]
Siberian Husky	USA	9035	0,007	Donner et al. [20]
Soft Coated Wheaten Terrier	USA	88	0.39	Zeng et al. [10]
Soft Coated Wheaten Terrier	USA	607	0,21	Donner et al. [20]
Spanish Water Dog	USA	96	0,073	Donner et al. [20]
Staffordshire Bull Terrier	USA	52	0.11	Zeng et al. [10]
Staffordshire Bull Terrier	USA	610	0,0016	Donner et al. [20]
Tamaskan (Aatu Tamaskan)	USA	59	0.18	Zeng et al. [10]
Tenterfield Terrier	USA	66	0.05	Zeng et al. [10]
Tibetan Terrier	USA	69	0.36	Zeng et al. [10]
Tibetan Terrier	USA	95	0,24	Donner et al. [20]
Treeing Walker Coonhound	USA	336	0,0045	Donner et al. [20]
Weimaraner	USA	647	0,0031	Donner et al. [20]
Welsh Terrier	USA	72	0.26	Zeng et al. [10]
West Highland White Terrier	USA	658	0.0007	Donner et al. [20]
White Swiss Shepherd	Italy	63	0.11	Ghilardi et al [24]
Wire Fox Terrier	USA	79	0.94	Zeng et al. [10]
Yorkshire Terrier	USA	8367	0,13	Donner et al. [20]
